# Uncovering the Kinematic Signature of Freezing of Gait in Parkinson’s Disease Through Wearable Inertial Sensors

**DOI:** 10.3390/s25165054

**Published:** 2025-08-14

**Authors:** Francesco Castelli Gattinara Di Zubiena, Alessandro Zampogna, Martina Patera, Giovanni Cusolito, Ludovica Apa, Ilaria Mileti, Antonio Cannuli, Antonio Suppa, Marco Paoloni, Zaccaria Del Prete, Eduardo Palermo

**Affiliations:** 1 Department of Mechanical and Aerospace Engineering, Sapienza University of Rome, 00184 Rome, Italy; francesco.castelligattinaradizubiena@uniroma1.it (F.C.G.D.Z.); ludovica.apa@uniroma1.it (L.A.); zaccaria.delprete@uniroma1.it (Z.D.P.); 2 Department of Human Neurosciences, Sapienza University of Rome, 00185 Rome, Italy; alessandro.zampogna@uniroma1.it (A.Z.); martina.patera@uniroma1.it (M.P.); antonio.suppa@uniroma1.it (A.S.); 3 IRCCS Neuromed, 86077 Pozzilli, Italy; 4 Department of Anatomical and Histological Sciences, Legal Medicine and Orthopedics, Sapienza University of Rome, 00185 Rome, Italy; giovanni.cusolito@uniroma1.it (G.C.); marco.paoloni@uniroma1.it (M.P.); 5 Department of Engineering, University Niccolò Cusano, 00166 Rome, Italy; ilaria.mileti@unicusano.it; 6 Department of Engineering, University of Messina, 98122 Messina, Italy

**Keywords:** wearable sensors, inertial measurement unit IMU, Parkinson’s disease (PD), spatiotemporal parameters, Freezing of Gait (FoG), gait analysis, biomechanics

## Abstract

Parkinson’s disease (PD) is a disorder that causes a decrease in motor skills. Among the symptoms that have been observed, the most significant is the occurrence of Freezing of Gait (FoG), which manifests as an abrupt cessation of walking. This study investigates the impact of spatiotemporal gait parameters using wearable inertial measurement units (IMUs). Notably, 30 PD patients (15 with FoG, 15 without) and 20 healthy controls were enrolled. Gait data were acquired using two foot-mounted IMUs and key parameters such as stride time, gait phase distribution, cadence, stride length, speed, and foot clearance were extracted. Results indicated a tangible decline in motor abilities in PD patients, especially in those with FoG. Differences were observed in the segmentation of gait phases, with diminished swing phase duration observed in patients, and in the diminished spatial parameters of stride length, velocity, and foot clearance. Additionally, to validate the results, the accuracy of IMU-derived clearance measurements was validated against an optoelectronic system. While the IMUs accurately detected maximum points, the minimum clearance showed a higher measurement error. These findings support the use of wearable IMUs as a reliable and low-cost alternative to laboratory systems for the assessment of gait abnormalities in PD. Moreover, they highlight the potential for early detection and monitoring of FoG in both clinical and home settings.

## 1. Introduction

Parkinson’s disease (PD) is a progressive neurodegenerative disorder characterized primarily by the degeneration of dopaminergic neurons within the substantia nigra pars compacta. This neuronal loss results in a marked reduction in dopamine synthesis and availability, disrupting basal ganglia circuits critical for motor control. Consequently, patients exhibit a spectrum of motor and non-motor symptoms [1]. Impaired ability to walk and maintain balance is among the most impactful motor disorders [1]. In PD patients, walking is often slow, with small, dragging steps, which impair the individual’s postural capacity and increase their risk of falling [1].

As the disease progresses, additional symptoms may occur. One of the most debilitating is Freezing of Gait (FoG), defined by sudden episodes of cessation or inability to start walking [2]. This phenomenon can occur in a variety of situations, such as at the beginning of the walking task, in overcoming narrow spaces or obstacles, during turning, or during dual-tasking [3,4]. This significantly impacts patients’ mobility, further increasing the risk of falling and reducing their quality of life [2,5]. Targeted rehabilitation interventions focusing on specific gait parameters have demonstrated potential to alleviate FoG in PD [6,7]. Thus, detailed kinematic characterization of gait in patients with FoG, compared to patients without FoG and healthy controls, is crucial for developing tailored rehabilitation strategies and objectively assessing treatment effectiveness [1].

The analysis of the duration and length of the stride, its elevation, speed, cadence, and distribution of the different phases of the stride can objectively assess patients’ motor impairment when compared to healthy individuals [8]. Measuring these parameters also allows for monitoring the progression of the disease, assessing the effectiveness of therapies, and developing more precise, patient-focused clinical treatment plans. The number of studies performing this quantitative analysis is rapidly growing, leveraging advanced approaches and technologies such as wearable sensors and machine learning to better understand the gait characteristics of Parkinsonian patients and FoG [8,9,10,11,12,13,14,15,16,17,18,19,20,21,22,23,24,25]. In 2024, Tang et al. [25] analyzed gait characteristics in Parkinsonian patients using wearable sensors. In their study, the researchers compared a group of PD patients who were exclusively in the OFF state of dopaminergic therapy, that is, in the absence of drug administration, with a sample of healthy individuals. The results showed reduced motor abilities of PD patients compared with healthy subjects. Although FoG was mentioned in the results, no specific data on patients who presented with the symptom were included. Additionally, among the spatiotemporal gait parameters measured, foot clearance, which plays a very important role in fall risk, is not present. Similarly, in 2017, Schlachetzki et al. [10] performed a gait analysis, using two inertial measurement units (IMUs) placed on the side of the foot, measuring several spatiotemporal parameters. In this instance, clearance was also measured, and it was found to have a reduced amplitude compared to the healthy control group. However, the study’s primary focus did not center on subjects with FoG.

Nevertheless, a few studies have made comparisons between patients with FoG and those without. In 2024, Lin et al. [21] employed a Kinect to ascertain the spatiotemporal parameters of patients with FoG, with the objective of evaluating their responsiveness to dopaminergic therapy. The findings indicated that patients with FoG exhibited a higher prevalence of motor complications and diminished parameters in comparison to subjects without FoG. This discrepancy is mitigated by the administration of the pharmaceutical agent. However, the issue of clearance remains unaddressed in their work. A comparable study by Zhao et al. [22] proceeded to analyze parameters specifically in the interictal period of FoG patients, obtaining similar results to those of Lin et al., again with no consideration for clearance.

The analysis of spatiotemporal parameters has been demonstrated to play a crucial role in the classification of patients with FoG, as evidenced by the studies conducted by [15,20]. Zampogna et al. [20] conducted a continuous analysis of PD patients in their homes for a period of approximately one week. This approach facilitated the acquisition of information regarding activities of daily living. The objective of this study was to differentiate between patients with and those without FoG. However, the results pertaining to spatiotemporal parameters demonstrated no statistically significant differences between the two groups. In contrast, Bouchouras et al. [15] employed a machine learning algorithm, a Random Forest, to differentiate between FoG subjects both in the OFF and ON states of therapy. The algorithm demonstrated a higher degree of efficacy in classifying FoG during the OFF state compared to the ON one. However, in both cases, the authors do not consider clearance in their analysis. This observation underscores a conspicuous need for the analysis of this parameter.

Furthermore, it is imperative to obtain validation of the systems employed that deviate from the gold standard, such as IMUs, to ensure the accuracy of the measurements made. This is particularly salient for parameters that are closely related to fall risk factors, such as clearance, which represents the elevation of the foot during ambulation. Despite the numerous validations conducted on spatiotemporal parameters associated with walking [26,27,28,29], to the best of the authors’ knowledge, clearance has not been a consideration in any of these studies. In 2024, He et al. [26] and, in 2025, Boutaayamou et al. [27] independently validated IMUs for measuring spatiotemporal parameters, excluding clearance, in healthy subjects. For patients with PD, similar research was conducted by Alberto et al. [29] in 2021, who validated a system comprising 15 IMUs for use in both supervised and unsupervised environments. Additionally, Yang et al. [28] in 2022 validated a system consisting of 5 IMUs for detecting early-stage PD during turns. Consistent with prior research on healthy individuals, neither study validated IMUs for foot clearance. Indeed, this parameter is frequently validated on systems using combinations of multiple techniques or sensors to maximize accuracy [18,30]. In 2017, Arami et al. [30] validated a clearance measurement system that used an IMU to support an infrared sensor for measuring clearance within a range of 4 to 30 cm. Conversely, the IMU’s primary function was to identify the foot inclination. In contrast, in 2024, Fehr et al. [18] validated a clearance measurement using a single IMU, tasked with measuring the parameter, combining it with a 3D scan of the foot that reconstructed its geometry. Consequently, this method enabled the identification of the lowest point of the foot during the swing phase. However, these studies do not utilize only a single IMU, a choice that would offer significant advantages in terms of comfort and simplicity when monitoring real-life situations. This has led to the necessity of investigating the accuracy of IMUs for clearance measurement.

This study describes the analysis of several spatiotemporal parameters obtained using IMUs, as an extension of a previous study [1], and pursues two main objectives.

The first objective is to assess motor function in individuals diagnosed with PD by comparing them with a healthy control group. Emphasis was placed on the phenomenon of FoG, with the subdivision of the patient sample into two distinct groups: experiencing and non-experiencing FoG as a PD symptom. An analysis of the differences in the parameters among healthy subjects, Parkinsonian subjects without FoG, and those with FoG was conducted to ascertain further motor impairments caused by this symptom. An experimental campaign of gait task acquisitions was conducted, both during the OFF state of therapy, i.e., in the absence of dopaminergic drug administration, and the ON state, i.e., during the effect of the dopaminergic drug. A series of spatiotemporal parameters was extracted from kinematic data acquired through IMUs and compared across different groups through a statistical study. This study aims to broaden knowledge regarding the phenomenon of FoG by including clearance, which is often not considered in overall analyses of spatiotemporal parameters. It also aims to make a comparison between different groups that include both healthy subjects and different classifications of PD, going on to distinguish between subjects with FoG and without FoG in OFF and ON states.

The second objective is to introduce and validate a methodology for measuring foot clearance using IMUs, building upon the parameter validation performed by Mileti et al. [24]. A comprehensive assessment of the accuracy of the IMU-based foot clearance estimation is provided, including a comparison with measurements obtained through a gold-standard optoelectronic motion capture system. This approach contributes to the validation of inertial systems by leveraging an IMU placed on the foot, thereby addressing the limitations identified in previous studies on spatiotemporal parameters. Additionally, it eliminates variables associated with the integration of additional sensors or systems that support the IMU, ensuring a more streamlined and comprehensive methodology.

## 2. Materials and Methods

### 2.1. Subjects

A total of 30 subjects diagnosed with PD were enrolled in this study (25 males and 5 females; mean age: 69.8 ± 8.9 years). Of these, 15 participants did not exhibit FoG (13 males and 2 females; mean age: 69.4 ± 9.8 years), while the remaining 15 participants demonstrated FoG (12 males and 3 females; mean age: 70.1 ± 8.2 years). The duration of the disease and its severity, as identified by clinical scales, were not subject to any restrictions. Additionally, patients who did not have overly debilitating gait changes were selected.

The control group consisted of 20 healthy individuals matched for age and sex distribution (14 males and 6 females; mean age: 69.9 ± 7.2 years) who were recruited. The number of subjects was selected following a preliminary power analysis. A one-way ANOVA and a Fisher’s exact test were performed to determine the homogeneity of the groups in terms of age and sex proportions.

Patients diagnosed with PD were evaluated both in the pharmacological OFF state, defined as at least 12 h after the withdrawal of dopaminergic therapy, and in the pharmacological ON state, assessed one hour after the administration of 150% of their usual L-Dopa dosage. The study was conducted according to the guidelines of the Declaration of Helsinki and was approved by the Ethics Committee of Sapienza University of Rome (protocol code 0372/2022 and date of approval 4 May 2022). Table 1 reports a summary of the demographic and clinical characteristics of the subjects.

### 2.2. Experimental Setup

**Figure 1:**
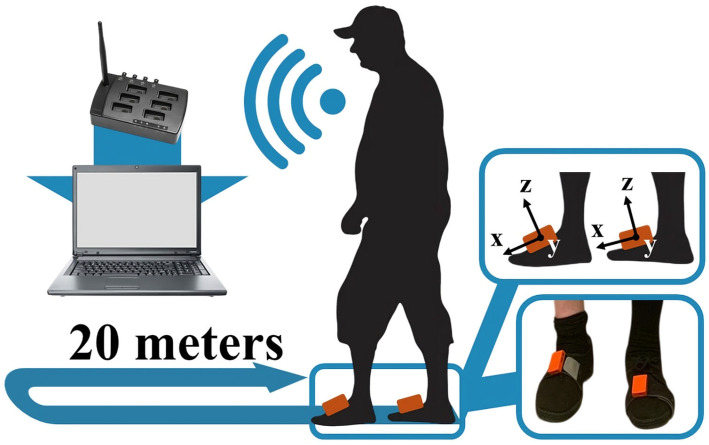
Schematic of the experimental setup. Foot-mounted IMUs, with their local axes, were used to collect gait data and transmitted signals wirelessly to the Awinda station connected to the acquisition computer.

The experimental setup involved the use of 2 MTw triaxial IMUs (Xsens, Movella Inc., Henderson, NV, USA) placed on the feet through elastic Velcro bands. Data from the IMUs were collected using an Awinda station connected to a PC, with a sampling rate of 100 Hz via the MT Manager 4.6 software (Movella Inc., Henderson, NV, USA). All tests were conducted along a straight, unobstructed corridor approximately 20 m in length, allowing the acquisition of 40 test strides per participant. A schematic representation of the experimental setup is provided in Figure 1.

### 2.3. Experimental Protocol

For PD patients, a brief interview and preliminary health assessments were conducted prior to testing, to evaluate their ability to safely perform the walking test in the OFF state. Only those who met the safety criteria proceeded with the full experimental protocol. This preliminary screening step was not necessary for healthy control subjects.

IMUs were positioned on the instep of each foot and affixed through elastic Velcro bands. Subsequently, the sensors were connected to the acquisition software via the Awinda station. The participant was escorted to the commencement of the corridor and requested to traverse it at a self-selected speed.

The test protocol entailed a direct trajectory traversing the entire 20 m length of the designated corridor. A visual marker was strategically placed at the end of the corridor to indicate the turning point. The subject was required to walk around this marker without interruption and subsequently return to the initial starting position. This mode resulted in a total straight walk length of approximately 40 m per subject. Only strides from the straight walk were utilized for analysis. For patients diagnosed with Parkinson’s disease, a physician was present to ensure their safety in the event of falls during the test. The experiment was replicated four times for each subject, who was requested to turn around twice to the left and twice to the right.

The trial was then replicated in a similar manner in the ON state of therapy, with a total of eight trials conducted for each patient. At the conclusion of each trial, data comprising accelerometer, gyroscope, magnetometer, and quaternion values were stored on the computer at a sampling rate of 100 Hz in a .txt format for subsequent analysis.

### 2.4. Data Analysis

The data collected from the IMUs were processed using MATLAB™ 2025a software (MathWorks Inc., Natick, MA, USA). Gyroscope data underwent filtration with a Butterworth filter of order 2, with a cutoff frequency of 3 Hz. Similarly, accelerometer data was filtered with a Butterworth filter of order 2, with a cutoff frequency of 10 Hz. Ten central strides of the straight walk were selected for going and returning, with a total of 20 strides per trial. This avoided issues related to acceleration and deceleration that occur during the commencement and cessation of the walk and the turning. To analyze the differences between patients with FoG and those without in their daily lives, regardless of the occurrence of the symptom, trials that presented FoG events were excluded from the analysis.

The following subsections delineate the methodology employed in deriving the spatiotemporal parameters examined in this study. For each spatiotemporal parameter, the mean and standard deviation were calculated for each of the following groups: healthy control (HC), overall parkinsonian patients in the OFF (PDoff) state, overall parkinsonian patients in the ON (PDon) state, parkinsonian patients without FoG in the OFF (NOFOGoff) state, parkinsonian patients without FoG in the ON (NOFOGon) state, parkinsonian patients with FoG in the OFF (FOGoff) state, and parkinsonian patients with FoG in the ON (FOGon) state.

#### 2.4.1. Stride Time Detection

**Figure 2:**
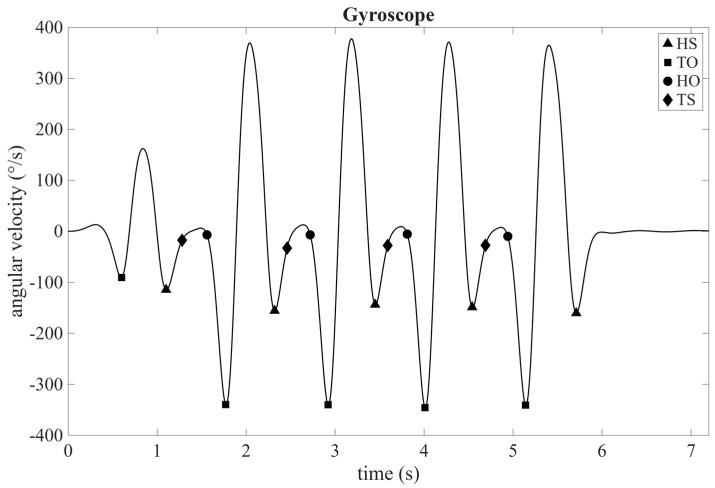
Representative angular velocity profile. Gait events of HS, TO, HO, and TS are reported as square, triangle, diamond, and circle, respectively.

Angular velocity in the sagittal plane (i.e., calculated on the body’s frontal axis, corresponding to the y-axis of the sensor) was used to identify key gait events, including heel strike (HS), toe strike (TS), heel off (HO), and toe off (TO). Following the approach described by Mileti et al. [31], HS and TO events were derived by identifying the second and first maximum points of a stride cycle, respectively. Meanwhile, TS and HO were derived by identifying the instants within the stance phase at which the absolute value of angular velocity crossed the 30°/s threshold. Accordingly, the stride time (ST) for each stride, calculated in seconds, could be derived by calculating the time elapsed between two consecutive HS events. Figure 2 shows a representative angular velocity trend, with the identified gait events marked.

#### 2.4.2. Gait Phases Detection

Based on the instants identified in the previous analysis, the different gait phases (GPs) were segmented. A four-phase model was adopted, including loading response (LR), defined as the interval between the HS and the subsequent TS; midstance (MS), defined as the flat foot (FF) period from TS to HO; pre-swing (PS), defined as the interval between HO and TO; and swing (SW), defined as the period between TO and the subsequent HS that initiates the next stride. Each phase was expressed as a percentage of the total stride time (ST) using the formula:

(1) $$\frac{t(\mathrm{GP})}{\mathrm{ST}}\cdot 100$$

where t(GP) identifies the time of the gait phase studied and ST is the total stride time.

#### 2.4.3. Cadence Detection

Cadence (CA) was calculated as the number of strides, determined from successive HS events by the total gait duration (in minutes). The result was rounded down to the nearest integer.

#### 2.4.4. Gait Phases Quality Index Detection

The Gait Phases Quality Index (GPQI) is an index initially introduced by Mileti et al. [32] that provides a summary quantification of gait quality. It quantifies the discrepancy between the subject’s GP sequence and a reference sequence obtained from the mean values of healthy subjects. GPQI was computed as

(2) $$\mathrm{GPQI}=\sqrt{\sum_{i=1}^{2}{\left({\mathrm{LR}}_{\mathrm{sub}}-{\mathrm{LR}}_{\mathrm{ref}}\right)}^{2}+{\left({\mathrm{MS}}_{\mathrm{sub}}-{\mathrm{MS}}_{\mathrm{ref}}\right)}^{2}+{\left({\mathrm{PS}}_{\mathrm{sub}}-{PS}_{\mathrm{ref}}\right)}^{2}+{\left({\mathrm{SW}}_{\mathrm{sub}}-{\mathrm{SW}}_{\mathrm{ref}}\right)}^{2}}$$

where i identifies the foot (right or left), ${\mathrm{LR}}_{\mathrm{sub}}$, ${\mathrm{MS}}_{\mathrm{sub}}$, ${\mathrm{PS}}_{\mathrm{sub}},$ and ${\mathrm{SW}}_{\mathrm{sub}}$ are the GPs of the subject, and ${\mathrm{LR}}_{\mathrm{ref}}$, ${\mathrm{MS}}_{\mathrm{ref}}$, ${\mathrm{PS}}_{\mathrm{ref}},$ and ${\mathrm{SW}}_{\mathrm{ref}}$ are the GPs of the reference sequence described previously in Section 2.4.2.

A lower GPQI indicates a gait phase distribution more similar to the reference, and therefore, higher gait quality.

#### 2.4.5. Stride Length Detection

The stride length (SL), in meters, was obtained by the Zero Velocity Update (ZUPT) algorithm. Initially, the acceleration data obtained from the accelerometer trials were rotated in the global coordinate system, and the component due to gravitational acceleration was removed. The rotation matrix was derived from the quaternions. Subsequently, the intervals relative to the flat foot, obtained by measuring the period between one TS and the subsequent HO, were set to zero. Then, the velocity was computed by integrating the acceleration on the three axes within the specified interval between HO and TS, employing the following formula:

(3) v(t)g=∫HOTSa(t)dtg

where vtg and a(t)g are the velocity and the acceleration in the global coordinate system. Subsequently, a linear detrend was implemented on the velocity to eradicate any drift that might have ensued from the integration of acceleration.

To obtain the subject’s stride length, the modulus of horizontal velocity was calculated with the following formula:

(4) $$v_{H}(t)=\sqrt{{v_{x}(t)}^{2}+{v_{y}(t)}^{2}}$$

where $v_{H}(t)$ is the horizontal velocity and $v_{x}(t)$ and $v_{y}(t)$ are the velocity components in the x and y axes belonging to the horizontal plane.

Once the horizontal velocity of the subject was obtained, the flat foot intervals were again set to zero and the integration of the horizontal velocity was repeated to obtain the horizontal displacement:

(5) $$d_{H(t)}=\int_{HO}^{TS}v_{H}(t)dt$$

where $d_{H}(t)$ is the horizontal displacement and $v_{H}(t)$ is the horizontal velocity.

The stride length is defined as the maximum value of the horizontal displacement obtained between two successive instants of HS.

**Figure 3:**
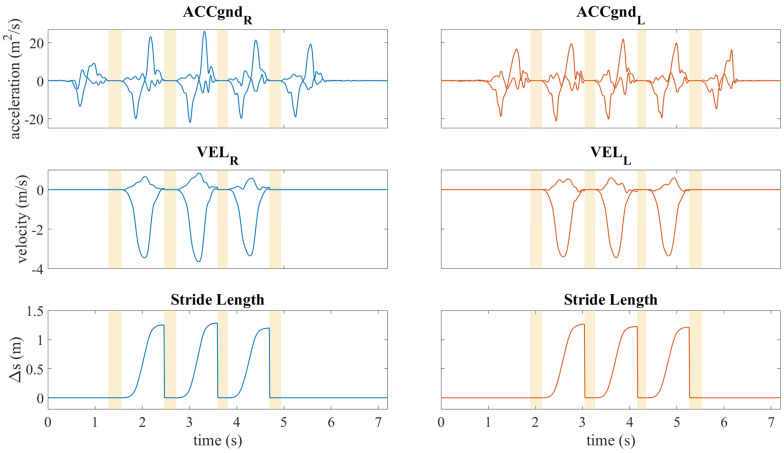
Illustration of the ZUPT algorithm applied to stride length estimation. The top panels illustrate the two horizontal components of acceleration in the global coordinate system for both feet (ACCgnd_R_ and ACCgnd_L_ are the accelerations of the right and left foot in the global coordinate system). The middle panels illustrate the horizontal components of velocity (VEL_R_ and VEL_L_ are the velocities of the right and left foot in the global coordinate system), while the bottom panels depict the horizontal displacement. Yellow-shaded regions indicate the flat foot intervals used in the ZUPT correction process.

An example of the process to obtain stride length is shown in Figure 3.

#### 2.4.6. Speed Detection

Walking speed (SP), calculated in m/s, was obtained by dividing the SL of each individual stride by the corresponding ST.

#### 2.4.7. Clearance Detection

The clearance (CL), calculated in mm, is defined as the vertical displacement of the foot in the sagittal plane. Its estimation followed a similar approach to that of SL, but only the vertical component of the acceleration signal was considered. Due to the unknown initial height of the foot from the ground, clearance was obtained as a relative vertical displacement trend.

**Figure 4:**
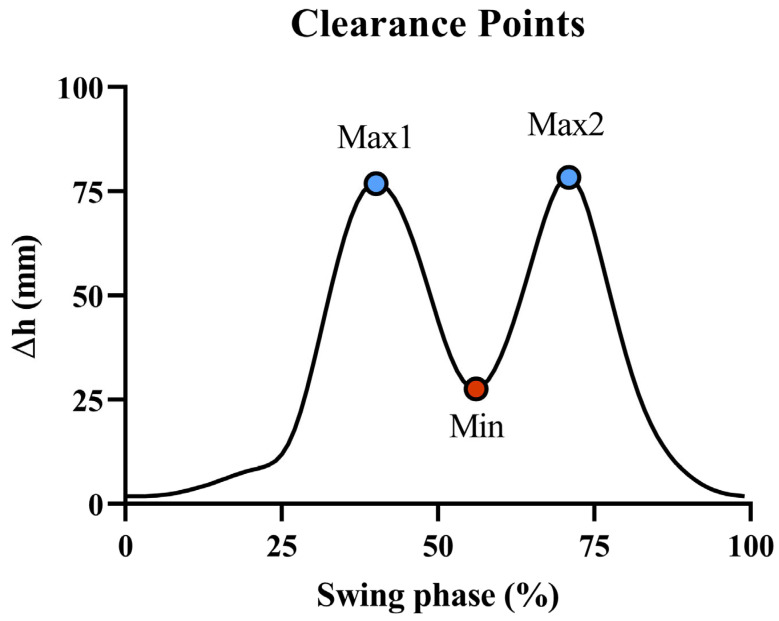
Example of foot clearance profile measured at the instep. Key points include the first local maximum (Max1), the second local maximum (Max2), and the local minimum (Min) between them.

The key parameters extracted from the CL signal were the first local maximum (Max1), the second local maximum (Max2), and the point of local minimum (Min) that lies between Max1 and Max2. These events represent the foot’s trajectory during swing. A graphical example is shown in Figure 4.

#### 2.4.8. Statistical Analysis

A statistical analysis was conducted to ascertain whether there were any substantial disparities in spatiotemporal parameters among the various groups. The groups compared are HC vs. PDoff, HC vs. PDon, HC vs. NOFOGoff, HC vs. NOFOGon, HC vs. FOGoff, HC vs. FOGon, NOFOGoff vs. FOGoff, and NOFOGon vs. FOGon.

Initially, the normality of the parameters was ascertained through a Shapiro-Wilk test. ST, GP, CA, GPQI, and SL exhibited non-normal distribution, while SP and CL (Max1, Max, and Min) demonstrated normal distribution. Subsequently, for parameters that did not conform to a normal distribution, a nonparametric Mann–Whitney U test was performed for each comparison, and the relative *p*-value was obtained. Conversely, for the determination of standard parameters, an independent-samples *t*-test was conducted for each comparison, yielding the respective *p*-value.

### 2.5. Clearance’s Accuracy Validation

The validation of the measurement of the spatial parameter of SL from IMU data has been previously addressed in the work of Mileti et al. [33]. In this study, we focused on validating the CL measurement using a similar methodology.

#### 2.5.1. Subjects

To validate the IMU-based CL estimation, a group of 3 healthy subjects (2 males and 1 female; mean age: 32 ± 8.2 years) was recruited. The sole stipulation was the absence of gait-related complications.

#### 2.5.2. Experimental Setup

The validation was executed using the same IMUs employed for spatiotemporal parameter analysis. A Vicon Nexus optoelectronic system (Vicon Motion Systems Ltd., Oxford, UK) with six Vicon IR Vero 2.2 cameras was utilized as a reference for validation. In the present study, inertial measurement units (IMUs) were strategically positioned on the plantar surfaces of both feet. Two reflective markers were placed on the lateral parts of the IMUs to follow the same vertical trajectory in the sagittal plane of the inertial unit. Calibration of the optoelectronic system yielded a capture volume with a surface area of 6 × 2 m. The data provided by the IMUs were acquired using the proprietary MVN Analyze Pro 2025.0 software, while the data from the cameras were acquired using the proprietary Vicon Nexus 2.17 software. The acquisitions were synchronized via a trigger cable.

#### 2.5.3. Experimental Protocol

Each subject performed a straight-line walk of approximately six meters, repeated for ten trials. During each trial, IMU data, including accelerometer, gyroscope, magnetometer, and quaternion signals, were recorded, along with the spatial coordinates of the reflective markers tracked by the optoelectronic system. All data were stored on a PC for post-processing in MATLAB 2025a.

#### 2.5.4. Data Analysis

**Figure 5:**
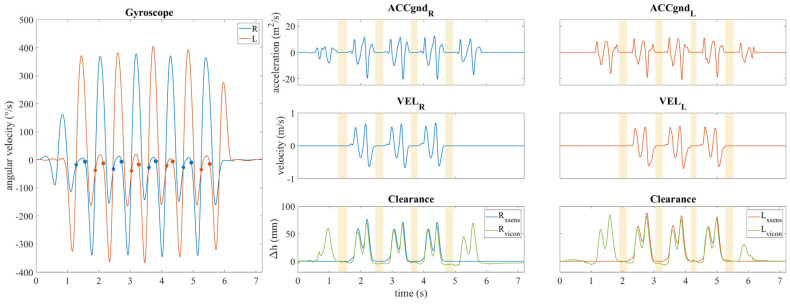
The subsequent illustration delineates the procedural framework of the ZUPT algorithm for clearance measurement. The left and right sensor angular velocity values are represented on the left panel, with diamonds indicating TS points and circles indicating HO points, which identify the flat foot intervals to be brought to zero. The top panels illustrate the vertical acceleration trends within the global reference system (ACCgnd_R_ and ACCgnd_L_ are the accelerations of the right and left foot in the global coordinate system). The middle panels illustrate the trends of the vertical component of velocity (VEL_R_ and VEL_L_ are the velocities of the right and left foot in the global coordinate system). The bottom panels illustrate the concurrent trends of clearance obtained from the IMUs and the optoelectronic system. Yellow bands indicate flat foot intervals used for correction.

The collected data were processed using MATLAB 2025a software. Vertical displacement of the foot-mounted IMUs was derived from the vertical component of acceleration in the global coordinate system. This estimation was performed using a Zero Velocity Update (ZUPT) algorithm, as previously described (Figure 5).

**Figure 6:**
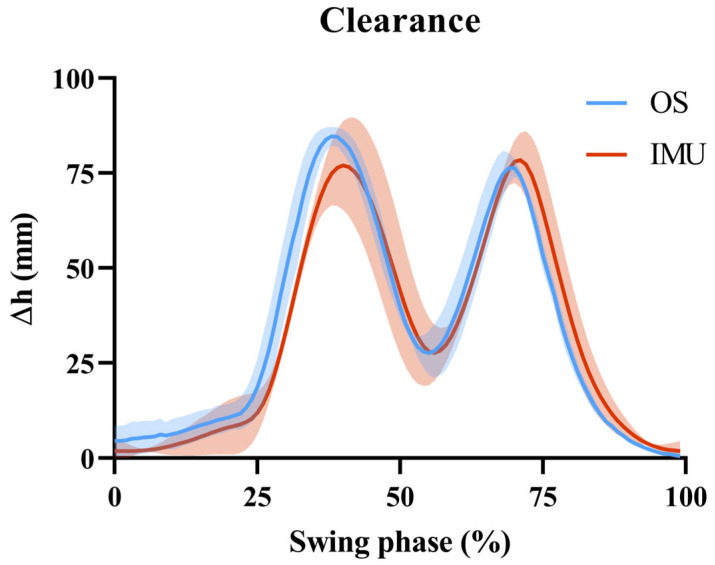
Comparison of average clearance profiles from the reference optoelectronic system (OS, in blue) and IMUs (IMU, in red), including standard deviations across trials for a representative subject.

To evaluate the accuracy of the IMU-based clearance measurements, individual strides were segmented and compared against those obtained from the optoelectronic system. Therefore, the clearance trend from the optoelectronic system was transformed into a displacement difference by subtracting its absolute minimum value from the vector. For each trial of each subject, three strides were extracted for a total of 30 strides per subject. Figure 6 presents an example of the average clearance trends obtained from the two systems for one subject.

Once the strides were divided, the clearance points of Max1, Max2, and Min were calculated for both systems. For each point, the absolute error and absolute relative percentage error were calculated according to the following formulas:

(6) eabs=xOScl−xIMUcl

(7) erel%=xOScl−xIMUclxOScl·100

where $e_{\mathrm{abs}}$ is the absolute error of the single stride, $e_{\mathrm{rel}\%}$ is the absolute relative percentage error, xOScl is the point of clearance measured by the optoelectronic system, and xIMUcl is the same point measured by the IMU.

Then the mean values of both errors were calculated for each subject among all strides. Finally, the mean absolute error and mean absolute relative percentage error among all subjects with relative standard deviations were calculated.

To assess the agreement between the two measurement methods, a Bland–Altman plot with a 95% confidence interval for each clearance parameter was generated.

## 3. Results

### 3.1. Spatiotemporal Parameters

**Figure 7:**
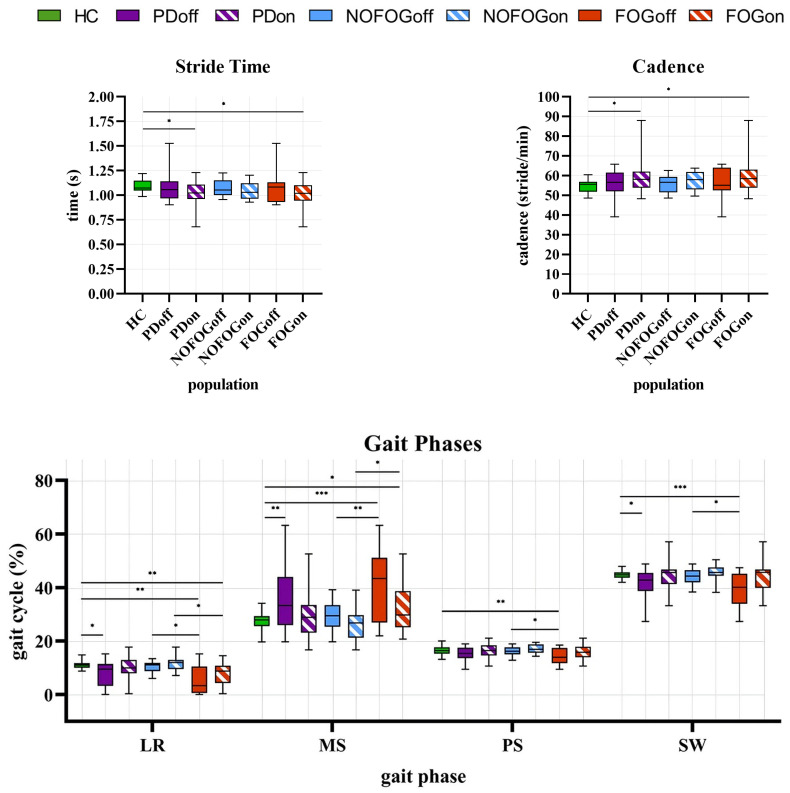

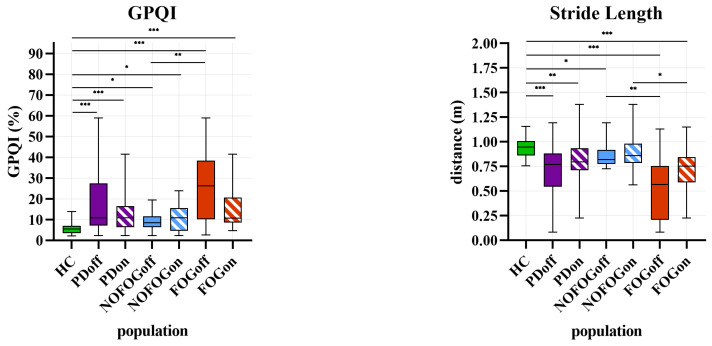
Group comparisons of non-normally distributed spatiotemporal gait parameters. Boxplots show median and 25th and 75th percentiles, as well as the maximum and minimum points. Statistical significance is indicated as follows: * *p* < 0.05, ** *p* < 0.01, *** *p* < 0.001.

**Figure 8:**
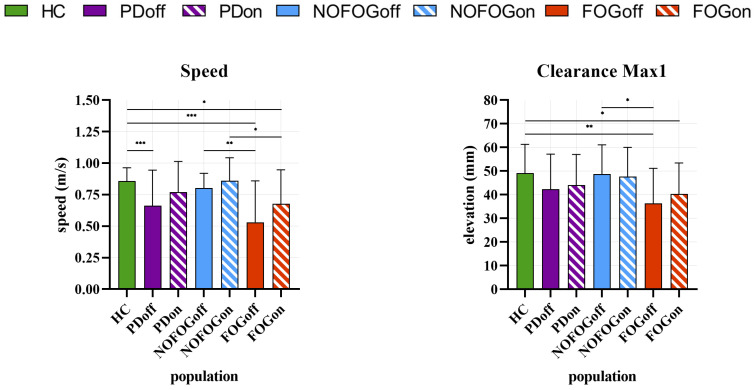

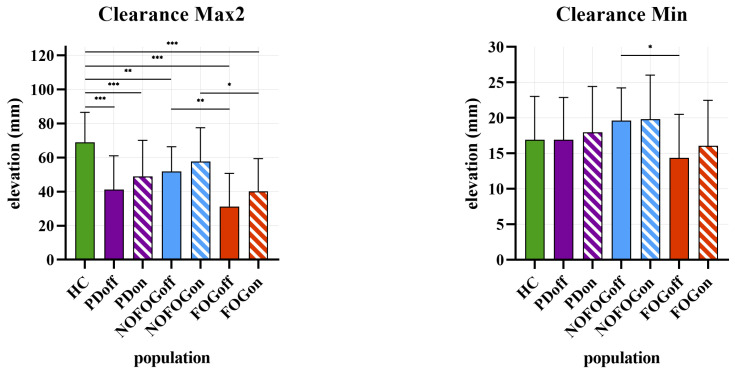
Group comparisons of normally distributed spatiotemporal gait parameters. Bar plots show the mean and the standard deviation. Statistical significance is indicated as follows: * *p* < 0.05, ** *p* < 0.01, *** *p* < 0.001.

As previously reported, a Mann–Whitney U test and an independent-samples *t*-test were used to compare all the groups studied, thereby enabling the analysis of spatiotemporal parameters. Figure 7 presents the nonparametric test’s boxplots, which include the median values and the 25th and 75th percentiles of the parameters for the groups as well as the maximum and minimum points, as well as the *p*-values for the various comparisons. Figure 8 presents the parametric test’s bar plots, which include the mean value and the standard deviation for the groups, as well as the *p*-values for the various comparisons. Table 2 reports a summary of the main results as well as the effect on the groups of the comparison.

#### 3.1.1. Stride Time

The Mann–Whitney U test revealed statistically significant differences, with *p* < 0.05, exclusively in the HC vs. PDon and HC vs. FOGon comparisons.

#### 3.1.2. Gait Phases

The Mann–Whitney U test revealed statistically significant differences, with *p* < 0.05 for LR and SW in HC vs. PDoff, for MS in HC vs. FOGon, for LR, PS, and SW in NOFOGoff vs. FOGoff, and for LR and MS in NOFOGon vs. FOGon; *p* < 0.01 for MS in HC vs. PDoff, for LR and PS in HC vs. FOGoff, for LR in HC vs. FOGon, and for MS in NOFOGoff vs. FOGoff; and *p* < 0.001 for MS and SW in HC vs. FOGoff.

#### 3.1.3. Cadence

The Mann–Whitney U test revealed statistically significant differences, with *p* < 0.05, exclusively in the HC vs. PDon and HC vs. FOGon comparisons.

#### 3.1.4. Gait Phase Quality Index

The Mann–Whitney U test revealed statistically significant differences, with *p* < 0.05 in HC vs. NOFOGoff and HC vs. NOFOGon; *p* < 0.01 in NOFOGoff vs. FOGoff; and *p* < 0.001 in HC vs. PDoff, HC vs. PDon, HC vs. FOGoff, and HC vs. FOGon.

#### 3.1.5. Stride Length

The Mann–Whitney U test revealed statistically significant differences, with *p* < 0.05 in HC vs. NOFOGoff and NOFOGon vs. FOGon; *p* < 0.01 in HC vs. PDon and NOFOGoff vs. FOGoff; and *p* < 0.001 in HC vs. PDoff, HC vs. FOGoff, and HC vs. FOGon.

#### 3.1.6. Speed

The *t*-test revealed statistically significant differences, with *p* < 0.05 in HC vs. FOGon and NOFOGon vs. FOGon; *p* < 0.01 in NOFOGoff vs. FOGoff; and *p* < 0.01 in HC vs. PDon and HC vs. FOGoff.

#### 3.1.7. Clearance

The *t*-test revealed statistically significant differences for Max1, with *p* < 0.05 in HC vs. FOGon and NOFOGoff vs. FOGoff, and *p* < 0.01 in HC vs. FOGoff.

It also revealed statistically significant differences for Max2, with *p* < 0.05 in NOFOGon vs. FOGon; *p* < 0.01 in HC vs. NOFOGoff and NOFOGoff vs. FOGoff; and *p* < 0.001 in HC vs. PDoff, HC vs. PDon, HC vs. FOGoff, and HC vs. FOGon.

As for Min, the only significant difference was found in NOFOGoff vs. FOGoff, with *p* < 0.05.

### 3.2. Clearance’s Accuracy Validation

**Figure 9:**
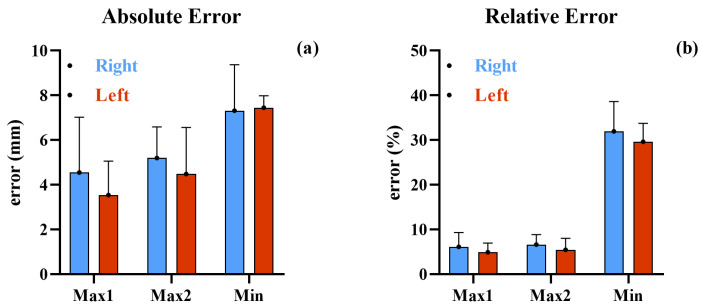
(**a**) Mean values and standard deviations of the absolute error among all subjects for the three points of Max1, Max2, and Min. (**b**) Mean values and standard deviations of the relative error among all subjects for same points.

Figure 9 presents the bar graphs of the mean absolute error and the mean absolute relative percentage error.

Absolute error is comparable between the two maximum points, but it is considerably greater for the minimum point. This discrepancy is further emphasized in the relative error analysis (Figure 9b), where the mean absolute relative percentage error for Min is significantly greater than for the maxima.

**Figure 10:**
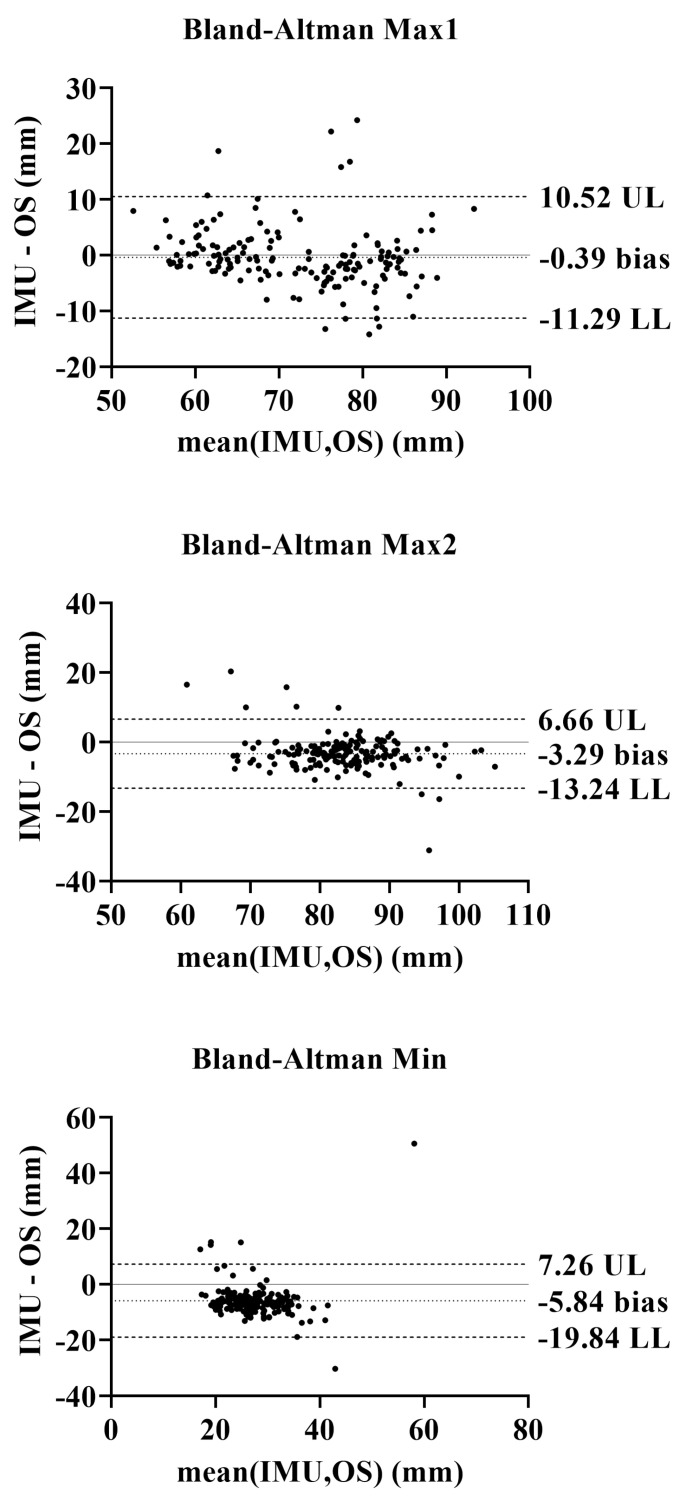
Bland–Altman plots comparing IMU and optoelectronic clearance measurements. Each plot shows mean bias and 95% limits of agreement (±1.96 SD) for Max1, Max2, and Min clearance points.

Figure 10 shows the Bland–Altman plots with a 95% confidence interval. The results indicate:

- Max1: Low bias and narrow limits of agreement, suggesting good consistency between methods.
- Max2: Slightly higher variability but still within acceptable bounds.
- Min: Larger bias and wider limits, indicating reduced reliability of IMU-based estimation for this parameter.

Overall, the IMUs demonstrated good accuracy in detecting maximum clearance points, with MARPE values below 10%, supporting their use in clinical and ambulatory settings. However, the estimation of minimum clearance remains challenging due to higher variability and systematic underestimation, warranting further algorithmic refinement.

## 4. Discussion

This section discusses the main findings of the study considering existing literature and clinical implications. The discussion is structured in two parts: the first focuses on the interpretation of spatiotemporal gait alterations observed in Parkinson’s disease (PD) and Freezing of Gait (FoG), while the second addresses the validation and clinical relevance of foot clearance estimation using wearable IMUs.

### 4.1. Interpretation of Spatiotemporal Gait Alterations in PD and FoG

Main findings reported in Table 2 can be summarized as follows:

- Significant differences in toe strike (TS) and cadence (CA) between healthy control (HC) and PD patients in the ON state. More pronounced alterations in FOG patients. No significant difference in the OFF state.
- Greater variability in gait phase organization is seen in PD, especially in FOG. Significant changes in loading response (LR), midstance (MS), and swing (SW) phases.
- GPQI is significantly higher in all PD groups vs. HC, especially in FOG groups.
- Significant reduction in stride length (SL) and speed (SP) in PD patients vs. HC. Most pronounced in FOG.
- Significant decrease in foot elevation (Max1, Max2) in PD, particularly severe in FOG.

Temporal parameters, such as stride time (ST) and cadence (CA), were generally consistent with previous literature [25,34,35]. Interestingly, significant differences in ST and CA were observed between healthy controls (HC) and PD patients in the ON state, particularly among those with FOG, in contrast with [36]. Contrary to expectations, no differences were found with the OFF state. This could be due to compensatory mechanisms implemented by PD patients, who normally have shorter strides and take more steps as a result. These alterations may be more prevalent in FOG patients as they are usually in a more advanced stage of the disease. The presence of these alterations in the ON state rather than the OFF state could be due to motor difficulties. In the OFF state, patients are unable to perform these less evident compensatory mechanisms.

Gait phase (GP) analysis revealed more nuanced differences. HC showed a well-defined and consistent phase distribution, while PD patients, especially those with FoG, exhibited greater variability, particularly in the midstance (MS) and swing (SW) phases, aligning with previous findings [25,35]. These alterations suggest a compensatory strategy aimed at increasing stability by prolonging the stance phase and reducing swing time. The comparison between NOFOGoff and FOGoff further confirmed the destabilizing effect of FoG, particularly during the LR and MS phases, which are critical for load transfer and balance. Notably, dopaminergic therapy partially restored gait phase organization in NOFOG patients, but had a limited effect in those with FoG, indicating a more persistent motor deficit in this subgroup.

These findings were further supported by the GPQI study, with values significantly elevated in all PD groups compared to HS, with the highest values observed in FoG patients.

Regarding spatial parameters, stride length (SL) and walking speed (SP) expectedly presented a marked reduction in PD subjects compared with HC, in accordance with [25,34] but with lower mean values than [21]. Particularly, the FOGoff group showed a significant reduction, consistent with the established phenomenon of hypokinesia in PD patients. It is noteworthy that drug therapy resulted in a reduction in the disparities between HC and PD, particularly among the NOFOGon group. However, this did not result in a complete restoration of performance in FoG subjects, where SL and SP remain significantly lower than in HC. This finding underscores the role of FoG in the deterioration of ambulatory performance, even in the presence of dopaminergic therapy.

The analysis of CL parameters, particularly Max1 and Max2, revealed substantial reductions in PDs, with the most pronounced deficits observed in the FOG group. Furthermore, discrepancies were identified between NOFOG and FOG in both OFF and ON states during the Max2 study. Max2 demonstrates substantial disparities across all comparisons between HC and the patient groups (PD, NOFOG, and FOG), except for the NOFOGon comparison, unveiling a pervasive deficiency in foot elevation capability during the latter half of the swing phase, in accordance with [10,37]. The reduction, particularly pronounced in patients with FoG, is crucial in terms of the increased risk of falling in PD patients. However, the minimum clearance does not exhibit substantial variation across the designated groups, except for NOFOGoff vs. FOGoff. This outcome is most likely attributable to the substantial variability inherent in the data sets of the respective groups under analysis. Furthermore, the limitations due to the extraction of spatial parameters from the double integration of the accelerometer signals must be taken into consideration, which, although compensated by the application of the ZUPT algorithm, still might lead to complications.

However, to ensure a comprehensive interpretation of the findings, it is important to acknowledge that the parameters under investigation did not consider any potential gait asymmetries. Indeed, no distinction was made between the right and left foot, as the emphasis was placed on evaluating the general disparities among the various groups, which could be more readily discerned and identified. This finding, however, may be indicative of greater or lesser variability between the groups, a matter that requires further investigation. It may also be of interest to evaluate the parameters by comparing them with the patients’ clinical scales to identify any patterns that could help in the early discrimination of the disease or its follow-up.

### 4.2. Validation of Clinical Relevance of Clearance Estimation

The validation study compared IMU-based clearance measurements to those from an optoelectronics system. While Max1 and Max2 showed good agreement and low bias, Min exhibited higher error and poor consistency, as evidenced by Bland–Altman plots and relative error metrics. These results suggest that IMU-based measurements are reliable for detecting maximum clearance points but less accurate for minimum clearance, a limitation that should be addressed in future algorithmic improvements.

Indeed, an examination of the two measurement methods reveals a negative average bias of approximately 5 mm, suggesting a substantial underestimation of IMUs in comparison to the optoelectronic system. The range of variability is also very high, which agrees with the analysis of absolute error and absolute relative percentage error values. This suggests that there is poor agreement between the two methods, and that the measurement of minimum clearance through IMU data is less reliable. The plots of Max1 and Max2 values provide more optimistic results, with smaller bias, especially for Max1, and variability ranges smaller than those of Min. Yet, the overall performance of IMUs in measuring spatiotemporal parameters is encouraging. Unlike many validation studies that omit clearance analysis [26,28,29] or rely on hybrid sensor systems [18,30], this study demonstrates that standalone IMUs can provide clinically relevant data, supporting their use as a viable alternative to laboratory-grade optoelectronic systems [10,25,30,37,38].

Importantly, the data indicate that FoG contributes to a generalized decline in motor function, even when freezing episodes are not actively triggered. This suggests that spatiotemporal gait parameters could serve as early indicators of FoG, aiding clinicians in diagnosis and treatment planning.

Future research should focus on expanding the sample size and improving cohort homogeneity in terms of gender, disease severity, and FoG status. Integrating additional modalities, such as electromyography, could offer deeper insights into neuro-muscular impairments. Moreover, the application of machine learning techniques for automated detection and prediction of FoG holds promise for enhancing fall risk assessment and personalized therapy.

## 5. Conclusions

The present study analyzed the spatiotemporal parameters in individuals with Parkinson’s disease (PD) using wearable inertial measurement units (IMUs), with a particular focus on the impact of Freezing of Gait (FoG). A cohort of 30 patients, divided into FoG and non-FoG subgroups, was analyzed under both OFF and ON dopaminergic therapy conditions and compared with 20 healthy controls. The results confirmed a significant deterioration in motor performance among PD patients, especially those with FoG. Notable impairments were observed in gait phase distribution, stride length, and foot clearance. While dopaminergic therapy improved several parameters, particularly in non-FoG patients, it did not fully restore gait performance in those with FoG, highlighting the persistent nature of this symptom. Interestingly, patients in the ON state exhibited unexpected differences in stride time and cadence compared to healthy controls, suggesting possible compensatory mechanisms. These findings warrant further investigation to clarify their clinical significance. Furthermore, possible gait asymmetry was not considered and therefore requires further study and could be addressed in future developments.

The validation of IMU-based clearance measurements demonstrated good accuracy for maximum clearance points (Max1 and Max2), with mean absolute relative errors below 10%. However, the estimation of minimum clearance proved less reliable due to higher variability and systematic underestimation. These results emphasize the need for further refinement of the signal processing algorithms used to extract clearance metrics from IMU data.

Overall, this study supports the use of wearable IMUs as a reliable, low-cost alternative to laboratory-based systems for gait analysis in PD, in consideration of the unlimited workspace. The findings also underscore the potential of IMU-derived parameters for early detection and monitoring of FoG, with implications for personalized treatment and fall prevention strategies. Future work should focus on expanding the sample size for spatiotemporal parameter analysis and for clearance validation, incorporating additional physiological signals (e.g., EMG), and exploring machine learning approaches for predictive modeling of FoG episodes.

## Figures and Tables

**Table 1:** Summary of the demographic and clinical characteristics of the subjects. Reported clinical scales are the Hoehn & Yahr (Y & H), the Unified Parkinson’s Disease Rating Scale part III (UPDRS-III), and the New Freezing of Gait Questionnaire (NFOG-Q). Since the FoG is not present, the NFOG-Q is not appliable (N/A) to the NOFOG group.

Group	NOFOG (Mean ± SD)	FOG (Mean ± SD)
Age (years)	69.4 ± 9.8	70.1 ± 8.2
Duration (years)	7.9 ± 3.0	8.1 ± 4.5
H&Y OFF	2.6 ± 0.5	3.1 ± 0.7
H&Y ON	2.1 ± 0.3	2.4 ± 0.6
UPDRS-III OFF	35.1 ± 10.6	48.0 ± 13.1
UPDRS-III ON	20.5 ± 9.4	28.1 ± 12.6
NFOG-Q	N/A	13.7 ± 3.3

**Table 2:** Summary of main results. Significant comparisons for different groups for each parameter and their effect on the pathological group are presented. Arrows in the column “Effect on the pathological group” represents the visible effect on the patient group in the specific comparison, indicating a decrease or increase in the affected parameter.

Parameter	Comparison	*p*-Value	Effect on the Pathological Group
Stride time (ST)	HC vs. PDon	<0.05	↓
	HC vs. FOGon	<0.05	↓
Gait phases (GP)	HC vs. PDoff	<0.05	LR ↓ SW ↓
	HC vs. PDoff	<0.01	MS ↑
	HC vs. FOGoff	<0.01	LR ↓ PS ↓
	HC vs. FOGoff	<0.001	MS ↑ SW ↓
	HS vs. FOGon	<0.05	MS ↑
	HS vs. FOGon	<0.01	LR ↓
	NOFOGoff vs. FOGoff	<0.05	LR ↓ PR ↓ SW ↓ (in FOGoff)
	NOFOGoff vs. FOGoff	<0.01	MS ↑ (in FOGoff)
	NOFOGon vs. FOGon	<0.05	LR ↓ MS ↑ (in FOGon)
Cadence (CA)	HC vs. PDon	<0.05	↑
	HC vs. FOGon	<0.05	↑
GPQI	HC vs. PDoff	<0.001	↑
	HC vs. PDon	<0.001	↑
	HC vs. NOFOGoff	<0.05	↑
	HC vs. NOFOGon	<0.05	↑
	HC vs. FOGoff	<0.001	↑
	HC vs. FOGon	<0.001	↑
	NOFOGoff vs. FOGoff	<0.01	↑ (in FOGoff)
Stride length (SL)	HC vs. PDoff	<0.001	↓
	HC vs. PDon	<0.01	↓
	HC vs. NOFOGoff	<0.05	↓
	HC vs. FOGoff	<0.001	↓
	HC vs. FOGon	<0.001	↓
	NOFOGoff vs. FOGoff	<0.01	↓ (in FOGoff)
	NOFOGon vs. FOGon	<0.05	↓ (in FOGon)
Speed (SP)	HC vs. PDoff	<0.001	↓
	HC vs. FOGoff	<0.001	↓
	HC vs. FOGon	<0.05	↓
	NOFOGoff vs. FOGoff	<0.01	↓ (in FOGoff)
	NOFOGon vs. FOGon	<0.05	↓ (in FOGon)
Clearance-Max 1	HC vs. FOGoff	<0.01	↓
	HC vs. FOGon	<0.05	↓
	NOFOGoff vs. FOGoff	<0.05	↓ (in FOGoff)
Clearance-Max 2	HC vs. PDoff	<0.001	↓
	HC vs. PDon	<0.001	↓
	HC vs. NOFOGoff	<0.01	↓
	HC vs. FOGoff	<0.001	↓
	HC vs. FOGon	<0.001	↓
	NOFOGoff vs. FOGoff	<0.01	↓ (in FOGoff)
	NOFOGon vs. FOGon	<0.05	↓ (in FOGoff)
Clearance-Min	NOFOGoff vs. FOGoff	<0.05	↓ (in FOGoff)

## Data Availability

The raw data supporting the conclusions of this article will be made available by the authors on request.

## Abbreviations

The following abbreviations are used in this manuscript:

PD	Parkinson’s Disease
FoG	Freezing of Gait
IMU	Inertial Measurement Unit
HS	Heel Strike
TS	Toe Strike
HO	Heel Off
TO	Toe Off
ST	Stride Time
GP	Gait Phases
LR	Loading Response
MS	Midstance
FF	Flat Foot
PS	Pre-swing
SW	Swing
CA	Cadence
GPQI	Gait Phases Quality Index
SL	Stride Length
ZUPT	Zero Velocity Update
SP	Speed
CL	Clearance
